# Alterations in Gut Microbiome Composition and Barrier Function Are Associated with Reproductive and Metabolic Defects in Women with Polycystic Ovary Syndrome (PCOS): A Pilot Study

**DOI:** 10.1371/journal.pone.0168390

**Published:** 2017-01-03

**Authors:** Lisa Lindheim, Mina Bashir, Julia Münzker, Christian Trummer, Verena Zachhuber, Bettina Leber, Angela Horvath, Thomas R. Pieber, Gregor Gorkiewicz, Vanessa Stadlbauer, Barbara Obermayer-Pietsch

**Affiliations:** 1 Department of Internal Medicine, Division of Endocrinology and Diabetology, Medical University Graz, Graz, Austria; 2 Department of Surgery, Division of Transplantation Surgery, Medical University Graz, Graz, Austria; 3 Center for Biomarker Research in Medicine (CBmed), Graz, Austria; 4 Department of Internal Medicine, Division of Gastroenterology and Hepatology, Medical University Graz, Graz, Austria; 5 Institute of Pathology, Medical University Graz, Graz, Austria; 6 BioTechMed, Interuniversity Cooperation, Graz, Austria; Peking University Third Hospital, CHINA

## Abstract

**Background:**

Polycystic ovary syndrome (PCOS) is a common female endocrinopathy of unclear origin characterized by hyperandrogenism, oligo-/anovulation, and ovarian cysts. Women with PCOS frequently display overweight, insulin resistance, and systemic low-grade inflammation. We hypothesized that endotoxemia resulting from a leaky gut is associated with inflammation, insulin resistance, fat accumulation, and hyperandrogenemia in PCOS. In this pilot study, we compared the stool microbiome, gut permeability, and inflammatory status of women with PCOS and healthy controls.

**Methods:**

16S rRNA gene amplicon sequencing was performed on stool samples from 24 PCOS patients and 19 healthy controls. Data processing and microbiome analysis were conducted in mothur and QIIME using different relative abundance cut-offs. Gut barrier integrity, endotoxemia, and inflammatory status were evaluated using serum and stool markers and associations with reproductive, metabolic, and anthropometric parameters were investigated.

**Results:**

The stool microbiome of PCOS patients showed a lower diversity and an altered phylogenetic composition compared to controls. We did not observe significant differences in any taxa with a relative abundance>1%. When looking at rare taxa, the relative abundance of bacteria from the phylum Tenericutes, the order ML615J-28 (phylum Tenericutes) and the family S24-7 (phylum Bacteroidetes) was significantly lower and associated with reproductive parameters in PCOS patients. Patients showed alterations in some, but not all markers of gut barrier function and endotoxemia.

**Conclusion:**

Patients with PCOS have a lower diversity and an altered phylogenetic profile in their stool microbiome, which is associated with clinical parameters. Gut barrier dysfunction and endotoxemia were not driving factors in this patient cohort, but may contribute to the clinical phenotype in certain PCOS patients.

## Introduction

Polycystic ovary syndrome (PCOS) is a common endocrine condition affecting women, which is characterized by androgen excess, oligo- or anovulation, and polycystic ovarian morphology. The reported worldwide prevalence of PCOS among women of reproductive age is 6–18% and varies due to the use of different diagnostic criteria, such as those issued by the National Institutes of Health (NIH) and the European Society of Human Reproduction and Embryology (ESHRE) [1]. PCOS phenotypes can range from mild to severe and are often accompanied by disorders of glucose and lipid metabolism[1–3]. Women with PCOS have a higher prevalence of infertility, pregnancy complications, depression, obesity, type 2 diabetes, and the related long-term outcomes [3–6]. Additionally, PCOS patients display a heightened inflammatory state with increased circulating C-reactive protein [7]. The etiology of PCOS has not been fully explained, but is assumed to be multifactorial, comprising genetics, the intrauterine environment, and lifestyle factors [3].

The gut microbiome is defined as the collective genomes of microorganisms that inhabit the gastrointestinal tract. Rodent studies have shown a direct effect of the gut microbiome on energy uptake and body weight, and gut microbiome changes are associated with obesity in humans [8–10]. In a noteworthy human study, stool transfer from healthy donors improved peripheral insulin sensitivity in patients with metabolic syndrome, suggesting a causal link between the gut microbiome and glucose metabolism [11]. Recent large-scale studies and meta-analyses support an association between the gut microbiome and obesity in humans, though this association may be weaker than previously assumed [12–14]. Furthermore, a link between gut bacteria, obesity, and host genetics is emerging, suggesting that certain bacteria predisposing to a healthy or unhealthy metabolic state may be heritable [13–15].

It has been shown in mice that gut bacteria can influence systemic metabolism via an alteration of the intestinal epithelial barrier, resulting in a translocation of bacterial endotoxin into the bloodstream [16]. This endotoxemia promotes insulin resistance and lipid storage through an up-regulation of pro-inflammatory signaling [17]. In 2012, Tremellen and Pearce postulated the hypothesis that diet-induced gut bacterial dysbiosis and subsequent gut barrier dysfunction and endotoxemia may drive the chronic inflammation and subsequent insulin resistance and androgen hypersecretion associated with PCOS [18]. To date, no published data on the gut microbiome in human PCOS patients exist, though there have been recent reports of alterations in the fecal microbiome in PCOS rodent models [19,20]. Data on gut permeability in PCOS patients are likewise scarce, though an increase in serum zonulin, a regulator of tight junction function, has been reported [21].

Considering the unclear knowledge of the etiology of PCOS and new insights from recent human and animal microbiome studies, we sought to investigate the gut microbiome in PCOS to assess its relevance in this condition. We aimed to describe the members and composition of the stool bacterial microbiome in a pilot cohort of PCOS patients and healthy controls. To investigate the hypothesis that the gut microbiome is associated with glucose, lipid, and steroid hormone metabolism and the translocation of bacterial products across the intestinal barrier, we assessed markers for gut barrier integrity, endotoxemia, and inflammation.

## Materials and Methods

A detailed description of the clinical and laboratory procedures can be found in S1 File. Detection limits and reliability indices of the used assays are provided in S1 Table.

### Study cohort

25 women with PCOS and 25 hormonally healthy controls, all premenopausal, were recruited at the endocrinological outpatient clinic of the University Hospital Graz from June-December 2014. PCOS was diagnosed according to the Rotterdam Criteria after exclusion of related disorders [2]. Exclusion criteria were the use of antibiotics, hormonal contraceptives, or antidiabetic medication within the preceding three months, gastrointestinal disease, active infections, a body mass index (BMI)<18, and smoking. All study participants were at least 18 years old and provided written informed consent. The study protocol was approved by the ethics committee of the Medical University Graz.

### Sampling

Study visits took place in the morning after an overnight fast. Anthropometric data were recorded. Following a baseline blood sampling, a two-hour, 75 g oral glucose tolerance test (oGTT; glucose, insulin) was performed.

A food frequency questionnaire designed by dieticians of the Clinical Medical Nutrition Therapy Unit, University Clinic Graz, was administered to assess the intake of major food groups (S2 File). Subjects were assigned to a carbohydrate- or animal protein-dominated diet type based on their responses.

Stool samples were collected using empty stool collection tubes with an inbuilt spatula (Praxisdienst GmbH, Longuich, Germany). Samples were stored short-term at -20°C (mean±SD = 4±1.1 days) and then at -70°C until further processing. There was no follow-up visit.

### Laboratory measurements

Serum estrone (E1), 17-estradiol (E2), total testosterone, androstenedione, dehydroepiandrosterone (DHEA), dehydroepiandrosterone sulfate (DHEAS), and dihydrotestosterone (DHT) were measured by liquid chromatography-tandem mass spectrometry at the Department of Clinical Chemistry at the University Hospital of South Manchester, Manchester, UK, as described by Keevil *et al*. [22–25].

Serum cortisol, thyroid-stimulating hormone (TSH), prolactin, insulin, anti-Muellerian hormone (AMH), and sex hormone-binding globulin (SHBG) were measured by automated chemiluminescence immunoassay. Serum luteinizing hormone (LH), follicle-stimulating hormone (FSH), 17-hydroxyprogesterone (17OH-P), high-sensitivity C-reactive protein (hs-CRP), interleukin-6 (IL-6), tumor necrosis factor-α (TNF-α), zonulin, diamine oxidase (DAO), lipopolysaccharide binding protein (LBP), soluble CD14 (sCD14), stool calprotectin, and stool zonulin were measured by enzyme-linked immunosorbent assay (ELISA). Plasma total cholesterol, high-density lipoprotein (HDL)-cholesterol, triglycerides, and glucose were measured by enzymatic colorimetric assay. A complete and differential blood count were performed. Serum endotoxin (= lipopolysaccharide, LPS) was measured using the HEK-Blue LPS Detection Kit (Invivogen, San Diego, USA) with an adapted protocol. Cells were cultured in 24-well plates (5x10^4^ cells/well). After 24 hours, medium was discarded and replaced with samples/endotoxin standards and detection medium. Cells were incubated for 21 hours at 37°C and color intensity was measured at a wavelength of 650 nm.

Adult-type hypolactasia (ATH) was determined by genotyping of the lactase-phlorizin hydrolase (*LCT*) gene polymorphism *LCT*-13910C>T [26]. DNA was isolated from whole blood using the NucleoSpin Blood kit (Machery-Nagel, Düren, Germany) followed by TaqMan SNP genotyping assay (Applied Biosystems, Waltham, USA).

Raw data of anthropometric, laboratory, and FFQ measurements can be found in S3 File.

### Calculations and definition of terms

BMI was calculated as $\frac{\text{Weight (kg)}}{{\text{Height (m)}}^{\text{2}}}$ and overweight defined as a BMI≥25. The homeostasis model assessment for insulin resistance (HOMA2-IR) index was calculated using the HOMA calculator V2.2.3 provided by the Diabetes Trial Unit, University of Oxford, UK (www.dtu.ox.ac.uk/homacalculator/, last accessed Dec 17, 2015) and insulin resistance defined as a HOMA2-IR≥2 [27]. The area under the curve (AUC) for glucose and insulin was calculated from the oGTT using the trapezoidal method. The free androgen index (FAI) was calculated according to the formula $100\times \frac{\text{Total testosterone (nmol/l)}}{\text{SHBG (nmol/l)}}$. Free testosterone and free DHT were calculated from total testosterone/DHT and SHBG according to Mazer, assuming a blood albumin concentration of 6.2 μmol/l [28].

### Next-generation sequencing

Total DNA was extracted from stool samples using the MagNA Pure LC DNA Isolation Kit III (Bacteria, Fungi) (Roche, Rotkreuz, Switzerland) according to the manufacturer’s instructions with additional bead-beating with MagNA Lyser Green Beads (Roche) and lysozyme treatment (Roth, Karlsruhe, Germany). One negative control consisting of 1x sterile phosphate buffered saline was included in each MagNA Pure run and subjected to all subsequent procedures.

The V1-2 region of the bacterial 16S rRNA gene was amplified using a polymerase chain reaction (PCR) and amplicons were sequenced on a MiSeq desktop sequencer (Illumina, Eindhoven, Netherlands) according to the manufacturer’s instructions. V1-2 was chosen as it provided a good balance between amplicon length (~350 bp) and overlap of forward and reverse reads (~150 bp).

Raw sequencing data are available in NCBI’s short read archive (SRA) under the accession number SRP077213.

### Sequencing data analysis

Raw reads were processed using the open-source software mothur V1.35.0 according to a published protocol (accessed April 2015) [29]. Open reference operational taxonomic unit (OTU) picking was performed in QIIME 1.8.0 using UCLUST against the greengenes 13.8 database [30–32]. An OTU was defined as a group of sequences with a similarity of 97% or more. Alpha rarefaction analyses were based on the number of observed OTUs and Faith’s phylogenetic diversity index and performed in QIIME. Principal coordinate analyses (PCoA) were based on unweighted and weighted UniFrac distances, calculated in QIIME, and plotted in GraphPad Prism 5 [33]. Taxa summaries were performed in QIIME. Three different OTU abundance cut-offs were tested to assess the impact of high- and low-abundance taxa and spurious OTUs resulting from sequencing errors. The cut-offs used were the removal of singleton OTUs, OTUs with a relative abundance <0.01%, and OTUs with a relative abundance <0.1%. Applying a relative abundance cut-off of 1% retained only 12 OTUs and was therefore deemed too strict.

### Real-time qPCR

The relative abundance of bacteria from the Tenericutes phylum was confirmed with real-time quantitative PCR using the primer set Ten662F and Ten862R, normalized to total 16S rRNA gene content using the primer set 926F and 1062R, as described by Yang *et al*. [34]. 20 ng DNA template were used in a 10 μl reaction on a LightCycler 480 Instrument (Roche) according to the manufacturer’s instructions.

### Statistical analysis

The statistical methods used in QIIME were: Nonparametric student’s t-tests using Monte Carlo permutations for alpha diversity comparisons, Mann-Whitney U tests for taxa comparisons, and Adonis for category comparisons of distance matrices. Benjamini-Hochberg false discovery rate (FDR) multiple testing correction was used for taxa comparisons.

All remaining statistical calculations were performed in IBM SPSS Statistics Version 22. Parametric or non-parametric tests were chosen based on the distribution of each variable. Unpaired t-tests and Mann-Whitney U tests were used for continuous data and Fisher’s Exact tests for categorical data. For correlation analyses, Pearson’s r is reported for parametric and Spearman’s rho (ρ) for nonparametric variables. In the case of missing values, patients were excluded from analysis for that particular variable. All data are expressed as median and interquartile range (IQR). A p-value<0.05 was considered statistically significant.

## Results

### Study subject characteristics

Of the 50 subjects included, all completed the baseline assessment and 49 returned a stool sample. Three controls were excluded due to previously undetected hyperandrogenemia, two subjects were excluded due to smoking, and one subject was excluded due to a BMI<18. The final analyses were performed with 20 healthy controls, of which 19 provided a stool sample, and 24 PCOS patients.

**Fig 1:**
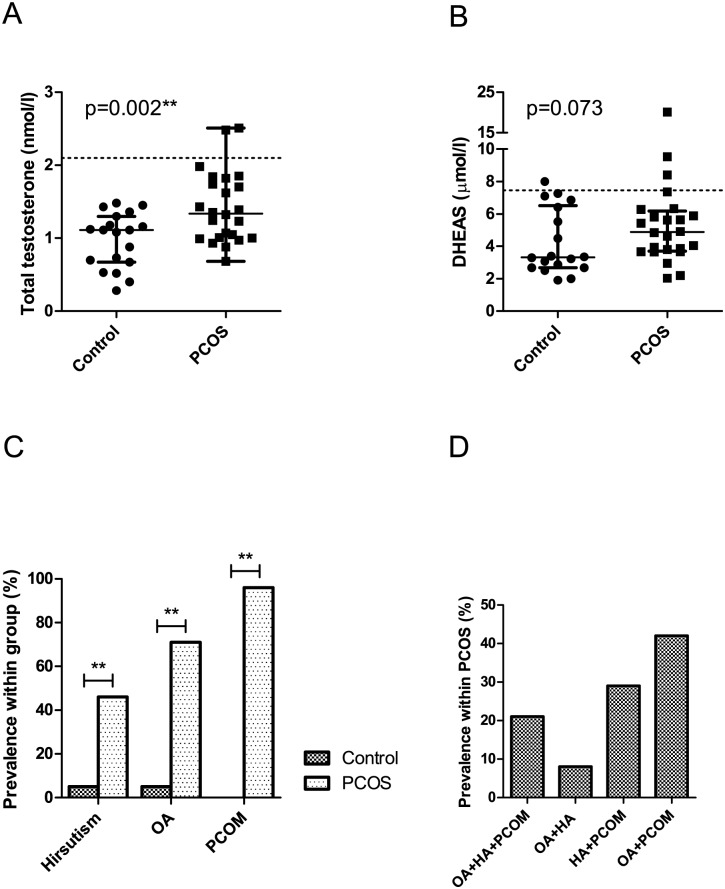
PCOS diagnostic criteria and phenotypes. Serum total testosterone (A) and DHEAS (B) levels in PCOS patients and controls. C. Prevalence of hirsutism, OA, and PCOM in PCOS patients and controls. D. Prevalence of phenotypes within PCOS patients according to the Rotterdam Criteria. PCOM: polycystic ovarian morphology, OA: oligo-/amenorrhoea, HA: hyperandrogenism (clinical and/or biochemical).

Study subject characteristics are summarized in Table 1 and Fig 1. PCOS patients fulfilled all three Rotterdam Criteria, displaying oligo-/amenorrhoea (OA, p<0.0001), hirsutism (p = 0.003), and polycystic ovarian morphology (PCOM, p<0.0001) accompanied by elevated AMH levels (p = 0.0002). However, serum androgen levels were only slightly higher than in the control group and median total testosterone values did not reach the predefined threshold for hyperandrogenemia (HA). Nevertheless, PCOS patients had significantly higher total testosterone (p = 0.002), androstenedione (p = 0.0003), and DHEA (p = 0.015) and lower E2 (p = 0.0004) than controls. Calculated free testosterone, free DHT, and FAI, which are considered more biologically relevant indicators of HA, were approximately 2–3 times higher in PCOS patients than in controls (p<0.0001 for all) [2]. PCOS patients also showed a typical increase in LH compared to controls (p = 0.035).

**Table 1:** Study subject characteristics.

	Reference range	Control (n = 20)		PCOS (n = 24)		
		median	IQR	median	IQR	p-value
Age		32	12.0	27	5.9	0.003**
Body mass index	18.5–25.0#	22.3	4.10	24.9	11.75	0.147
Waist to hip ratio	< 0.85#	0.80	0.063	0.82	0.077	0.439
Fasting glucose (mmol/l)	< 7.0†	4.5	0.50	4.7	0.59	0.209
2h glucose (mmol/l)	< 11.1†	4.3	1.09	4.8	1.15	0.296
AUC glucose (mmolh/l)	§	10.2	4.52	10.9	3.61	0.273
Fasting insulin (pmol/l)	20.9–173.8	41.4	51.08	84.4	55.25	0.022*
2h insulin (pmol/l)	§	129	140.0	188	336.7	0.371
AUC insulin (mmolh/l)	§	353	427.3	691	562.0	0.009**
HOMA2-IR	< 2.0	0.8	1.05	1.7	1.20	0.027*
Total cholesterol (mmol/l)	< 5.2	4.6	0.64	4.5	1.13	0.699
HDL-cholesterol (mmol/l)	> 1.0	2.0	0.42	1.7	0.49	0.006**
Triglycerides (mmol/l)	< 1.65	0.59	0.248	0.74	0.242	0.010*
Follicle-stimulating hormone (IU/l)	0.5–61.2‡	9.2	8.11	7.5	2.73	0.178
Luteinizing hormone (IU/l)	2.0–22.0‡	5.8	9.34	9.3	8.60	0.042*
LH:FSH ratio	§	1.2	1.19	1.5	1.06	0.035*
Anti-Muellerian hormone (pmol/l)	1.4–65.2	26.8	22.42	61.1	52.59	0.0002***
Total testosterone (nmol/l)	0.37–2.1	1.1	0.56	1.3	0.77	0.002**
Dihydrotestosterone (nmol/l)	§	0.34	0.241	0.46	0.528	0.096
Androstenedione (nmol/l)	0.89–7.5	2.6	1.61	4.2	2.69	0.0003***
Dehydroepiandrosterone (nmol/l)	§	13.7	11.37	21.4	12.40	0.015*
Dehydroepiandrosterone sulfate (μmol/l)	§	3.3	3.74	4.9	2.35	0.073
Estrone (pmol/l)	§	274	184.8	195	118.9	0.138
17-Estradiol (pmol/l)	§	436	285.8	163	181.1	0.0005***
Free androgen index	§	1.3	0.68	3.1	2.75	<0.0001***
Free testosterone (pmol/l)	§	10.6	5.86	20.9	13.00	<0.0001***
Free dihydrotestosterone (pmol/l)	§	1.3	1.03	3.0	2.19	<0.0001***
		**# of cases**	**% of cases**	**# of cases**	**% of cases**	**p-value**
Polycystic ovarian morphology		0	0	22	96	<0.0001***
Hirsutism		1	5	11	46	0.003**
Oligo-/Amenorrhoea		1	5	17	71	<0.0001***
High carbohydrate diet		8	40	9	38	0.555
High animal protein diet		12	60	15	63	0.555
Adult-type hypolactasia		5	25	5	21	0.511

AUC: area under the curve; HOMA2-IR: homeostasis model assessment for insulin resistance; HDL: high-density lipoprotein.

^#^according to the World Health Organization,

^†^according to the American Diabetes Association,

^‡^depending on menstrual cycle stage,

^§^reference range not defined.

An increased basal insulin secretion (p = 0.022) and AUCinsulin (p = 0.009) in the oGTT, elevated total triglycerides (p = 0.010), and reduced HDL-cholesterol (p = 0.006) were observed in the PCOS group. BMI did not differ between PCOS patients and controls (p = 0.147). PCOS patients were significantly younger than controls (p = 0.003), with median ages of 27 and 32, respectively.

When classifying PCOS patients based on the four possible phenotypes under the Rotterdam Criteria, the majority (42%) of patients displayed the phenotype D (OA+PCOM), while 21 and 8% displayed the “classical” phenotypes A (OA+HA+PCOM) and B (OA+HA), respectively (Fig 1). Type C (HA+PCOM) was represented with a prevalence of 29%.

### Characterizing the stool microbiome in PCOS

The total number of reads analyzed and OTUs identified at each cut-off level are presented in S2 Table. The number of reads analyzed did not differ between PCOS and control samples, indicating comparable and adequate sequencing coverage. As expected, applying relative abundance filters substantially reduced the number of observed OTUs while causing only a modest decrease in the number of reads analyzed.

The OTU relative abundance followed a characteristic long-tailed distribution in the singleton-removed and 0.01% filtered data (S1 Fig). The curve of the 0.1% filtered data lacked the long tail, indicating that at this threshold, a large proportion of true low-abundant OTUs were filtered out. The 0.01% filtered curve appeared similar to the singleton-removed data, while including much fewer OTUs, indicating that at this threshold a good balance between filtering out sequencing errors and retaining the original community structure of the sample was achieved. Therefore, the subsequent analyses were conducted using the 0.01%-filtered data.

**Fig 2:**
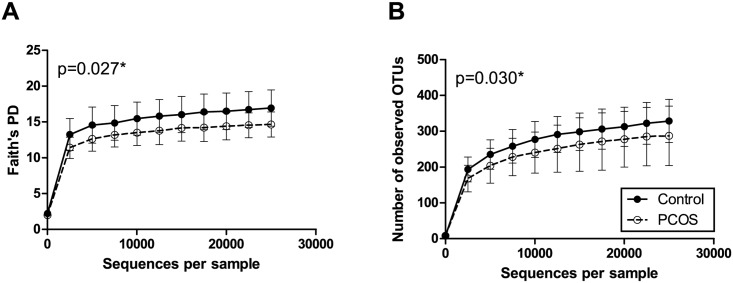
Alpha rarefaction curves of stool samples from PCOS patients and controls. Faith's phylogenetic diversity (A) and the number of observed OTUs (B). All samples were rarefied to the smallest observed number of reads (24,991). Median and IQR are plotted.

In alpha diversity analyses, PCOS patients exhibited an approximately 15% lower Faith’s phylogenetic diversity and number of observed OTUs than controls (p = 0.027 and 0.030, respectively) (Fig 2). Alpha diversity curves showed adequate saturation at the selected rarefaction level.

**Fig 3:**
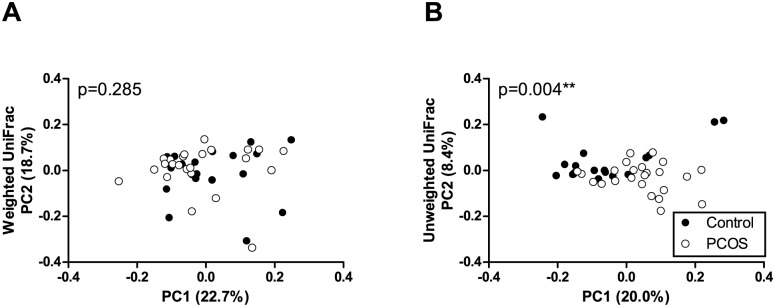
Principal coordinate analysis (PCoA) plots of stool samples from PCOS patients and controls. PCoA plots of weighted (A) and unweighted (B) UniFrac distance matrices. Each dot represents the bacterial community composition of one individual stool sample. Axis titles indicate the percentage variation explained.

In beta diversity analysis, which compares samples based on overall bacterial community composition, a statistically significant clustering of samples from PCOS patients and controls was observed in unweighted, but not weighted UniFrac analysis (Fig 3). Unweighted UniFrac only takes into account OTU presence/absence data and does not place emphasis on bacterial abundances. In weighted UniFrac analysis, where more abundant OTUs have a stronger effect on the result, no difference was observed between patients and controls.

Stool microbiome communities were dominated by the phyla Firmicutes and Bacteroidetes, with Bacteroides as the single most abundant genus (Table 2). No statistically significant differences were observed between PCOS patients and controls in bacterial taxa with a relative abundance >1% or in the Firmicutes:Bacteroidetes ratio.

**Table 2:** Relative abundances of bacterial genera and phyla with a median relative abundance >1%.

	% of total bacteria in Control (n = 19)		% of total bacteria in PCOS (n = 24)		
**Most abundant genera (>1%)**	**median**	**IQR**	**median**	**IQR**	**FDR p-value**
†Bacteroides	30.1	16.87	38.6	14.78	0.701
#Ruminococcaceae unclass. 1	6.9	3.76	6.6	4.45	0.879
#Lachnospiraceae unclass. 1	5.6	3.45	6.4	4.48	0.669
#Clostridiales unclass. 1	4.6	2.71	3.7	2.43	0.669
#Lachnospiraceae unclass. 2	2.8	2.77	3.8	2.57	0.693
#Clostridiales unclass. 2	3.6	3.81	2.7	2.57	0.087
†Rikenellaceae unclass.	2.5	5.25	3.1	2.55	0.970
#Faecalibacterium	2.3	2.19	3.5	3.12	0.651
#Dialister	1.5	4.27	1.6	4.08	0.693
†Parabacteroides	1.9	2.38	1.2	1.45	0.916
#Ruminococcus	2.6	2.18	0.4	1.48	0.087
†[Barnesiellaceae] unclass.	1.0	2.26	1.1	1.57	0.942
#Ruminococcaceae unclass. 2	1.4	1.66	0.9	3.07	0.697
#Erysipelotrichaceae unclass.	0.9	0.64	1.2	0.69	0.151
‡Sutterella	1.6	1.58	0.6	1.20	0.347
**Most abundant phyla (>1%)**	**median**	**IQR**	**median**	**IQR**	**FDR p-value**
†Bacteroidetes	48.0	9.49	50.8	13.64	0.980
#Firmicutes	48.1	12.13	47.6	11.76	0.867
‡Proteobacteria	2.5	3.15	1.5	1.94	0.229
F:B ratio	1.0	0.34	0.9	0.49	0.922

Unclass.: unclassified, the lowest classified taxonomic level is shown, FDR: Benjamini Hochberg false discovery rate correction. Square brackets indicate a greengenes suggested taxonomic assignment.

^#^Firmicutes,

^†^Bacteroidetes,

^‡^Proteobacteria.

**Fig 4:**
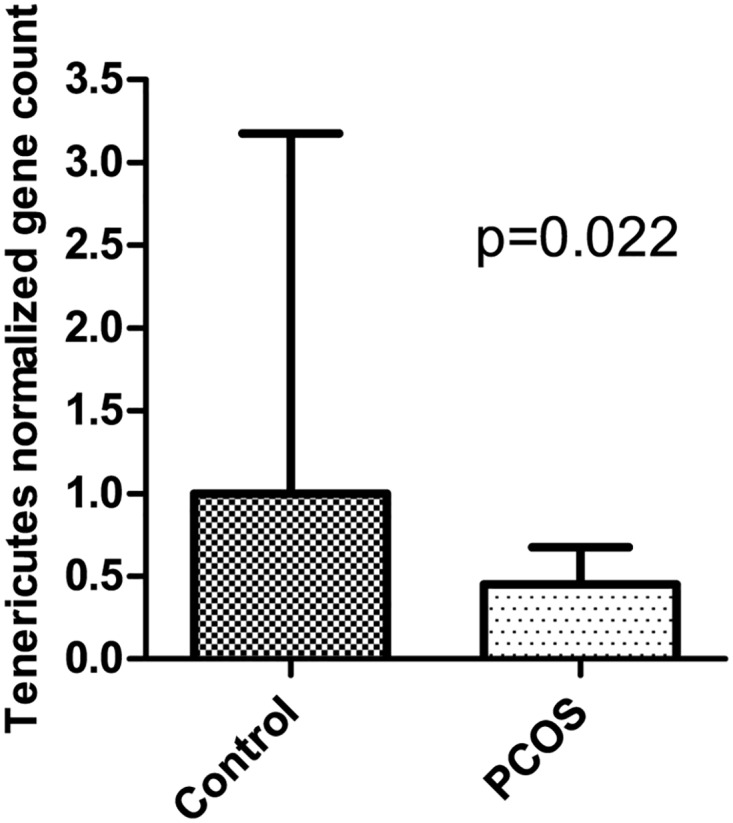
Relative abundance of Tenericutes in fecal samples from PCOS patients and controls assessed by real-time qPCR. Ct values were normalized to total 16S rRNA content. Median and IQR are plotted.

When comparing rare taxa (relative abundance <1%), bacteria from the phylum Tenericutes showed a significantly lower relative abundance in PCOS patients (p<0.0001) (Table 3). This finding was confirmed by real-time qPCR, where PCOS Tenericutes DNA content was more than two-fold lower in PCOS patients than in controls (p = 0.022) (Fig 4). This result became more significant after removing one outlier with very high Tenericutes counts in the control group (p = 0.003, data not shown). One unclassified genus from this phylum belonging to the order ML615J-28, was less abundant in PCOS patients (p = 0.026). An unclassified genus from the phylum Bacteroidetes and the family S24-7 was also less abundant in PCOS patients (p = 0.039). While bacteria from these taxa were present at a low relative abundance in a number of control samples, they were undetectable in most PCOS samples. Bacteria from the phylum Tenericutes were detected with 10 or more reads in 11/19 (58%) of controls and 1/24 (4%) of PCOS patients. S24-7 were detected with 10 or more reads in 9/19 (47%) of control samples and 1/24 (4%) of PCOS samples and ML615J-28 in 5/19 (26%) of control samples and 0/24 (0%) of PCOS samples. Negative controls did not contain sequences from any of the taxa that were found to be differentially abundant in PCOS patients and controls (S3 Table).

**Table 3:** Relative abundances of bacterial genera and phyla that differed significantly between PCOS patients and controls.

	% of total bacteria in Control (n = 19)		% of total bacteria in PCOS (n = 24)		
**Differentially abundant genera**	**median**	**IQR**	**median**	**IQR**	**FDR p-value**
#S24-7 unclass.	0.000	1.1473	0.000	0.0000	0.039*
†ML615J-28 unclass.	0.006	0.0344	0.000	0.0000	0.026*
**Differentially abundant phyla**	**median**	**IQR**	**median**	**IQR**	**FDR p-value**
†Tenericutes	0.019	0.0835	0.000	0.0000	<0.0001***

Unclass.: unclassified, the lowest classified taxonomic level is shown, FDR: Benjamini-Hochberg false discovery rate correction.

^#^Bacteroidetes,

^†^Tenericutes.

**Fig 5:**
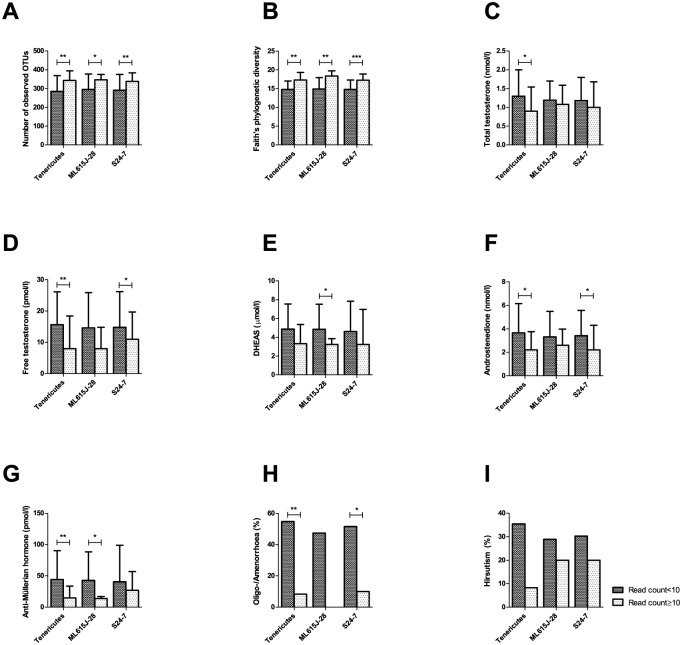
Association of differentially abundant taxa with bacterial diversity and reproductive parameters. The number of samples with detectable levels of the relevant taxa (read count ≥10) was 12/43 for Tenericutes, 5/43 for ML615J-28, and 10/43 for S24-7. Median and IQR are plotted for continuous variables.

All three differentially abundant taxa were positively associated with bacterial diversity (Fig 5A and 5B). Probands with detectable levels of these bacteria showed lower serum androgens and AMH and a reduced prevalence of oligo-amenorrhoea (Fig 5C–5H), while there was no association with hirsutism (Fig 5I).

To investigate a possible confounding effect of age, overweight, insulin resistance, diet type, and hypolactasia, diversity analyses were repeated with these variables. Age was found to be positively associated with the abundance of the three significant taxa and positively and weakly with Faith’s phylogenetic diversity, but not the number of observed OTUs or weighted or unweighted UniFrac distance matrices (S2 Fig). The remaining studied parameters did not influence alpha or beta diversity results (S3 and S4 Figs) or relative abundances at any taxonomic level, with one exception. A diet high in carbohydrates was associated with a higher abundance of bacteria from the phylum Cyanobacteria (S2 Fig).

### Gut barrier integrity and inflammation in PCOS

**Fig 6:**
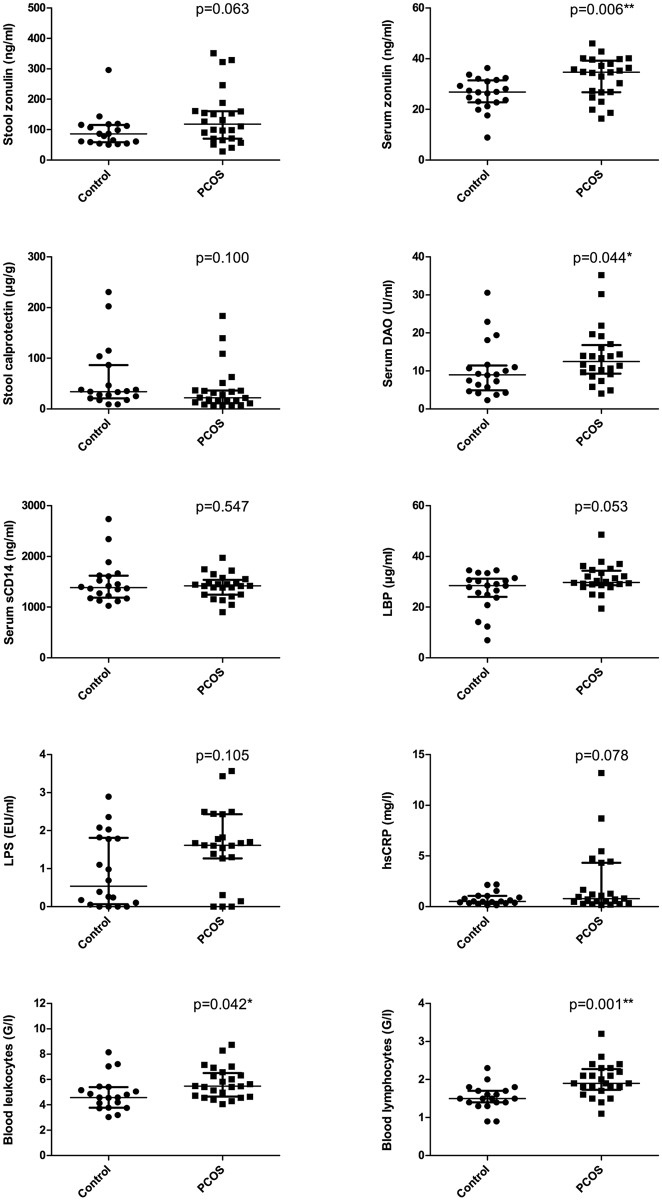
Markers of gut barrier integrity and inflammation in PCOS patients and controls. DAO: diamine oxidase, sCD14: soluble CD14 (endotoxin co-receptor), LBP: lipopolysaccharide binding protein, LPS: lipopolysaccharide (= endotoxin), hsCRP: high-sensitivity C-reactive protein. Dots represent individual study subjects. Bars represent median and IQR.

Zonulin, a regulator of tight junction function, was significantly higher in the serum (p = 0.006), but not in the stool (p = 0.063) of PCOS patients (Fig 6). Serum DAO, a marker for intestinal epithelial damage, was significantly higher in PCOS patients (p = 0.044). Serum endotoxin was not different between PCOS patients and controls. LBP, which binds endotoxin, tended to be higher in the serum of PCOS patients (p = 0.053), while the soluble LPS receptor, sCD14, was unchanged. Stool calprotectin, a marker for intestinal inflammation, was unchanged between patients and controls.

In the assessment of systemic inflammatory status, PCOS patients had significantly higher blood leukocytes and lymphocytes than controls (p = 0.042 and 0.001, respectively) (Fig 6). Serum hsCRP was not significantly different from control levels (p = 0.078). TNF-α and IL-6 were undetectable in the majority of serum samples (data not shown).

**Fig 7:**
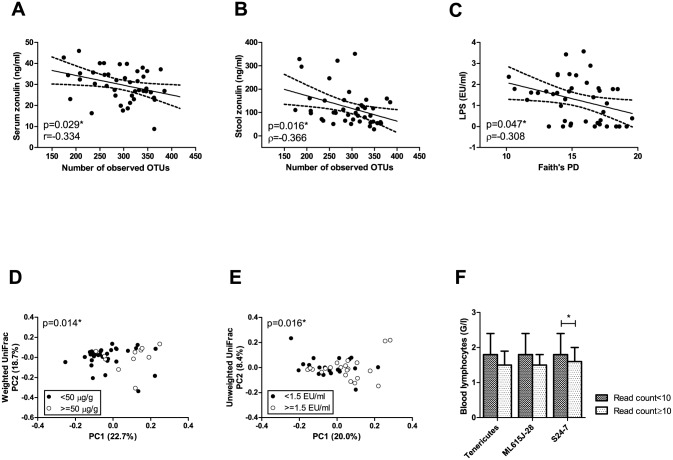
Association of microbiome parameters with parameters of gut barrier integrity and inflammation. LPS: lipopolysaccharide (= endotoxin), hsCRP: high-sensitivity C-reactive protein. Dots represent individual study subjects. Median and IQR are plotted. The number of samples with detectable levels of the relevant taxa (read count ≥10) was 12/43 for Tenericutes, 5/43 for ML615J-28, and 10/43 for S24-7.

We investigated the association of microbial alpha and beta diversity and Tenericutes, ML615J-28, and S24-7 with parameters of gut barrier function and inflammation. Stool zonulin, serum zonulin, and LPS were inversely and moderately associated with indices of alpha diversity (Fig 7A–7C). Serum LPS and stool calprotectin were associated with sample clustering in unweighted and weighted UniFrac plots, respectively (Fig 7D and 7E). Total blood lymphocyte counts were inversely associated with Tenericutes relative abundance (Fig 7F). Serum hsCRP, DAO, LBP, and blood leukocytes were not associated with alpha or beta diversity or the relative abundance of Tenericutes, ML615J-28, or S24-7 (data not shown).

## Discussion

In this pilot study, we provide a first description of the stool microbiome of women with PCOS diagnosed according to the Rotterdam criteria. We observed a statistically significant 15% reduction in Faith’s phylogenetic diversity and the number of observed OTUs, accompanied by phylogenetic microbiome profile shifts between samples from PCOS patients and controls in unweighted UniFrac analysis. Additionally, PCOS patients had a lower relative abundance of three bacterial taxa, the phylum Tenericutes, the order ML615J-28 (belonging to the phylum Tenericutes), and the family S24-7 (belonging to the phylum Bacteroidetes).

Our results are in accordance with a recent study investigating the fecal microbiome in a letrozole-induced mouse model of PCOS, which reported an approximately 8% decrease in Faith’s phylogenetic diversity in letrozole-treated compared to vehicle-treated mice [19]. In addition, differences in several bacterial taxa, including a decreased relative abundance of several OTUs from the family S24-7 in letrozole-treated mice, were found. In another recent study using a letrozole-induced rat model of PCOS, treatment with either lactobacillus or fecal transplantation from healthy rats improved reproductive abnormalities [20]. The authors also reported changes in fecal microbiome profiles, but did not use next-generation sequencing and targeted only a small selection of bacterial taxa with qPCR.

While these are currently the only studies investigating the fecal microbiome in PCOS, there are a number of studies on the gut microbiome in obesity and insulin resistance. A reduction in stool bacterial diversity of up to 30% has previously been reported in individuals with obesity and metabolic dysfunction [10,35]. Recent meta-analyses have concluded that there is a significant, yet very modest, decrease in alpha diversity in obesity, while differences in community composition and specific taxa are highly study-dependent and do not show a statistically significant overarching pattern [12,36]. In patients with type 2 diabetes, a reduction of putatively beneficial butyrate-producing bacteria has been reported, while alpha diversity was found to be unchanged [37–39]. We did not observe significant differences in alpha diversity, UniFrac distance matrices, or taxa relative abundances due to body mass index or insulin sensitivity in our study. This may have been due to the small sample size of our cohort, as well as the pilot design, which was not conceived to test the association between the gut microbiome and obesity or insulin resistance. The significant changes that we did observe may result from another variable specific to PCOS or, more likely, from a combination of variables which may include BMI and insulin resistance.

There are some previous reports of associations between the significant taxa in our study and metabolic health. Lim *et al*. found higher diversity and an enrichment of bacteria from the phylum Tenericutes in healthy individuals compared to metabolic syndrome patients [14]. Interestingly, Tenericutes were identified as a significantly heritable taxon among the healthy group. In another study of microbiome heritability, the order ML615J-28 was identified as part of a heritable bacterial cluster associated with leanness in humans [14,15]. In another study, the relative abundance of bacteria from the family S24-7 was decreased in mice receiving a high-fat diet and increased after exercise [40]. We conclude that although the PCOS patients in our study had a relatively mild metabolic phenotype with only slightly higher indices of insulin resistance and dyslipidemia than the control group and no significant difference in BMI, these disturbances may nevertheless be reflected in the gut microbiome of these patients. Furthermore, as both PCOS and the gut microbiome appear to have some degree of heritability, these mechanisms may be linked. Hypothetically, the same genetic factors predisposing to PCOS may also promote the establishment of a metabolically adverse gut microbiome, which would further drive the PCOS phenotype.

The PCOS patients in our cohort displayed all typical symptoms of the disorder, but surprisingly few had pronounced hyperandrogenemia. The median total testosterone and DHEAS levels were well below the defined cut-offs in the majority of patients. This resulted in 42% of patients with the Rotterdam phenotype D (OA and PCOM), which is greater than previously published data from larger PCOS cohorts and may therefore not be an accurate representation of the general PCOS population [41]. The phenotype D has been reported to have less reproductive dysfunction and metabolic risk than the remaining phenotypes [41]. Therefore, the high prevalence of this “mild” phenotype may have attenuated some microbiome changes that are present in the more symptomatic phenotypes. The small sample size and pilot design of our study precluded a statistical analysis of the different subgroups. However, we found associations between microbiome parameters and serum androgens and oligo-/amenorrhoea.

As this was a pilot study, we included only a small number of subjects in each group. While there are currently no general recommendations for sample sizes in microbiome studies, it has been estimated that an “n” of 20 per group allows for the detection of differences with effect sizes of 0.008 in unweighted and 0.04 in weighted pairwise distances with 90% power [42]. While our sample size was appropriate for unweighted UniFrac analysis, the study was underpowered to detect small effects in weighted UniFrac analysis, and likely also in taxa comparisons. Conversely, the effects due to the gut microbiome in our mildly affected cohort may be too small to be detected even in a larger sample of the same phenotypes. We therefore hypothesize that more pronounced microbiome changes will be observed when investigating a larger cohort including more severe PCOS phenotypes with obesity (BMI>30) and manifest type 2 diabetes, as well as more pronounced hyperandrogenemia. We cannot exclude that the observed microbiome changes are the result of biological changes due to aging, as controls were significantly older than PCOS patients and age was associated with phylogenetic diversity and the relative abundance of the three significant taxa. However, as both groups were premenopausal and the difference in the median ages was only five years, we argue that any age effect would be much weaker than effects due to PCOS or other factors. It has been reported that PCOS symptoms can be more pronounced in young patients and attenuated with age [43]. We therefore hypothesize that there is a gradient of gut dysbiosis in PCOS, with younger, more symptomatic patients showing greater microbiome changes.

Bacteria from the taxa Tenericutes, ML615J-28, and S24-7 were present at a very low relative abundance, representing a median of 0–0.02% of all bacteria in controls. As the included negative controls did not contain any sequences belonging to these taxa, they are likely not the result of a contamination. That such rare taxa can have a biologically significant impact on the host is unlikely, but not impossible. For example, segmented filamentous bacteria (SFB) are found in very low abundance or not at all in mouse feces, but are highly abundant in the small intestinal mucosa and play a crucial role in early-life immune system maturation [44]. In humans, an age-related decline in stool SFB abundance has been observed and SFB are rarely detected in adult stool [45]. If some of the taxa which were reduced in our PCOS patients are heritable, as was suggested recently for ML615J-28 and Tenericutes, they may regulate hormonal and metabolic development in the small or proximal large intestine in early life up to puberty and decline in abundance thereafter [14,15].

We found significant or borderline significant increases in several parameters of intestinal barrier dysfunction and inflammation in PCOS patients. Zonulin is a regulator of tight junction function in humans and serum levels correlate with *in vivo* gut permeability assessed by lactulose/mannitol test [46]. The serum zonulin increase observed in our study is in accordance with recent studies in PCOS patients and obese individuals, where serum zonulin was correlated with measures of insulin resistance and fecal bacterial colony count [21,47]. Serum endotoxin and hsCRP did not differ significantly between patients and controls. Zonulin, calprotectin, LPS, and blood lymphocytes were associated with gut microbiome parameters, indicating support for the gut barrier-endotoxemia-inflammation mechanism hypothesized by Tremellen and Pearce and previously shown in mice by Cani *et al*. [16–18]. However, this study does not provide enough evidence to make the claim that this process is driving PCOS symptoms. Similar to the gut microbiome, gut barrier changes may become more pronounced in specific PCOS phenotypes, such as those meeting all three Rotterdam criteria or with more pronounced insulin resistance and/or obesity.

Strengths of our study are the thorough metabolic and reproductive characterization of our PCOS patients, including androgen measurements by mass spectrometry, the use of relative abundance filters to reduce OTU inflation due to sequencing errors, and the consideration of possible confounding factors such as age, diet, and adult-type hypolactasia. Weaknesses of the study include the small sample size, which precluded stratification of PCOS patients based on diagnostic criteria or metabolic parameters, the high prevalence of PCOS patients with a mild phenotype, and the assessment of gut barrier function using surrogate parameters rather than an in vivo lactulose-mannitol test. Despite these shortcomings, the study achieved its aim of providing a first descriptive look into the gut microbiome-gut barrier axis in PCOS and a knowledge base for future studies.

## Supporting Information

S1 Fig Cumulative distribution of OTU relative abundances at different cut-off levels. (JPG)

S2 Fig Association of age and diet type with microbiome parameters. (JPG)

S3 Fig Alpha rarefaction curves of possible confounders. ATH: adult-type hypolactasia.
(JPG)

S4 Fig Principal coordinate analysis (PCoA) plots of possible confounders. ATH: adult-type hypolactasia.
(JPG)

S1 File Supplementary Materials and Methods. (PDF)

S2 File Food frequency questionnaire. (PDF)

S3 File Anthropometric, laboratory, and FFQ raw data. (XLSX)

S1 Table Detection limits and reliability indices of used assays. (DOCX)

S2 Table Overview of sequencing data. (DOCX)

S3 Table Contaminations. (DOCX)

## References

[1] March WA, Moore VM, Willson KJ, Phillips DI, Norman RJ, Davies MJ. The prevalence of polycystic ovary syndrome in a community sample assessed under contrasting diagnostic criteria. Hum Reprod. 2010;25(2): 544–51. 10.1093/humrep/dep399 19910321

[2] Rotterdam ESHRE/ASRM-Sponsored PCOS consensus Workshop Group. Revised 2003 consensus on diagnostic criteria and long-term health risks related to polycystic ovary syndrome (PCOS). Hum Reprod. 2004;19(1): 41–7. 14688154 10.1093/humrep/deh098

[3] Dumesic DA, Oberfield SE, Stener-Victorin E, Marshall JC, Laven JS, Legro RS. Scientific Statement on the Diagnostic Criteria, Epidemiology, Pathophysiology, and Molecular Genetics of Polycystic Ovary Syndrome. Endocr Rev. 2015;36(5): 487–525. 10.1210/er.2015-1018 26426951 PMC4591526

[4] Wehr E, Gruber HJ, Giuliani A, Moller R, Pieber TR, Obermayer-Pietsch B. The lipid accumulation product is associated with impaired glucose tolerance in PCOS women. J Clin Endocrinol Metab. 2011;96(6): E986–90. 10.1210/jc.2011-0031 21470992

[5] Lerchbaum E, Schwetz V, Giuliani A, Obermayer-Pietsch B. Assessment of glucose metabolism in polycystic ovary syndrome: HbA1c or fasting glucose compared with the oral glucose tolerance test as a screening method. Hum Reprod. 2013;28(9): 2537–44. 10.1093/humrep/det255 23756702

[6] Kollmann M, Klaritsch P, Martins WP, Guenther F, Schneider V, Herzog SA, et al Maternal and neonatal outcomes in pregnant women with PCOS: comparison of different diagnostic definitions. Hum Reprod. 2015;30(10): 2396–403. 10.1093/humrep/dev187 26223675

[7] Escobar-Morreale HF, Luque-Ramirez M, Gonzalez F. Circulating inflammatory markers in polycystic ovary syndrome: a systematic review and metaanalysis. Fertil Steril. 2011;95(3): 1042–8. 10.1016/j.fertnstert.2010.11.036 21168133 PMC3079565

[8] Backhed F, Ding H, Wang T, Hooper L V, Koh GY, Nagy A, et al The gut microbiota as an environmental factor that regulates fat storage. Proc Natl Acad Sci U S A. 2004;101(44): 15718–23. 10.1073/pnas.0407076101 15505215 PMC524219

[9] Turnbaugh PJ, Ley RE, Mahowald MA, Magrini V, Mardis ER, Gordon JI. An obesity-associated gut microbiome with increased capacity for energy harvest. Nature. 2006;444(7122): 1027–31. 10.1038/nature05414 17183312

[10] Turnbaugh PJ, Hamady M, Yatsunenko T, Cantarel BL, Duncan A, Ley RE, et al A core gut microbiome in obese and lean twins. Nature. 2009;457(7228): 480–4. 10.1038/nature07540 19043404 PMC2677729

[11] Vrieze A, Van Nood E, Holleman F, Salojarvi J, Kootte RS, Bartelsman JF, et al Transfer of intestinal microbiota from lean donors increases insulin sensitivity in individuals with metabolic syndrome. Gastroenterology. 2012;143(4): 913–6.e7. 10.1053/j.gastro.2012.06.031 22728514

[12] Sze MA, Schloss PD. Looking for a Signal in the Noise: Revisiting Obesity and the Microbiome. MBio. 2016;7(4). 10.1128/mBio.01018-16 PMC4999546 27555308

[13] Beaumont M, Goodrich JK, Jackson MA, Yet I, Davenport ER, Vieira-Silva S, et al Heritable components of the human fecal microbiome are associated with visceral fat. Genome Biol. 17(1): 189 10.1186/s13059-016-1052-7 27666579 PMC5036307

[14] Lim MY, You HJ, Yoon HS, Kwon B, Lee JY, Lee S, et al The effect of heritability and host genetics on the gut microbiota and metabolic syndrome. Gut. 2016; gutjnl– 2015–311326. 10.1136/gutjnl-2015-311326 27053630

[15] Goodrich JK, Waters JL, Poole AC, Sutter JL, Koren O, Blekhman R, et al Human genetics shape the gut microbiome. Cell. 2014;159(4): 789–99. 10.1016/j.cell.2014.09.053 25417156 PMC4255478

[16] Cani PD, Bibiloni R, Knauf C, Waget A, Neyrinck AM, Delzenne NM, et al Changes in gut microbiota control metabolic endotoxemia-induced inflammation in high-fat diet-induced obesity and diabetes in mice. Diabetes. 2008;57(6): 1470–81. 10.2337/db07-1403 18305141

[17] Cani PD, Amar J, Iglesias MA, Poggi M, Knauf C, Bastelica D, et al Metabolic endotoxemia initiates obesity and insulin resistance. Diabetes. 2007;56(7): 1761–72. 10.2337/db06-1491 17456850

[18] Tremellen K, Pearce K. Dysbiosis of Gut Microbiota (DOGMA)—a novel theory for the development of Polycystic Ovarian Syndrome. Med Hypotheses. 2012;79(1): 104–12. 10.1016/j.mehy.2012.04.016 22543078

[19] Kelley ST, Skarra D V, Rivera AJ, Thackray VG. The Gut Microbiome Is Altered in a Letrozole-Induced Mouse Model of Polycystic Ovary Syndrome. PLoS One. 2016;11(1): e0146509 10.1371/journal.pone.0146509 26731268 PMC4701222

[20] Guo Y, Qi Y, Yang X, Zhao L, Wen S, Liu Y, et al Association between Polycystic Ovary Syndrome and Gut Microbiota. PLoS One. 2016;11(4): e0153196 10.1371/journal.pone.0153196 27093642 PMC4836746

[21] Zhang D, Zhang L, Yue F, Zheng Y, Russell R. Serum zonulin is elevated in women with polycystic ovary syndrome and correlates with insulin resistance and severity of anovulation. Eur J Endocrinol. 2015;172(1): 29–36. 10.1530/EJE-14-0589 25336505

[22] Owen LJ, Wu FC, Keevil BG. A rapid direct assay for the routine measurement of oestradiol and oestrone by liquid chromatography tandem mass spectrometry. Ann Clin Biochem. 2014;51(Pt 3): 360–7. 10.1177/0004563213501478 24084694

[23] Owen LJ, Wu F, Buttler R, Keevil BG. ANNALS EXPRESS: A direct assay for the routine measurement of testosterone, androstenedione, dihydrotestosterone and dehydroepiandrosterone by LC-MS/MS. Ann Clin Biochem. 2015;53(Pt 5): 580–7. 10.1177/0004563215621096 26589631

[24] Munzker J, Hofer D, Trummer C, Ulbing M, Harger A, Pieber T, et al Testosterone to dihydrotestosterone ratio as a new biomarker for an adverse metabolic phenotype in the polycystic ovary syndrome. J Clin Endocrinol Metab. 2015;100(2): 653–60. 10.1210/jc.2014-2523 25387259

[25] Chadwick CA, Owen LJ, Keevil BG. Development of a method for the measurement of dehydroepiandrosterone sulphate by liquid chromatography-tandem mass spectrometry. Ann Clin Biochem. 2005;42(Pt 6): 468–74. 10.1258/000456305774538175 16259799

[26] Mattar R, de Campos Mazo DF, Carrilho FJ. Lactose intolerance: diagnosis, genetic, and clinical factors. Clin Exp Gastroenterol. 2012;5: 113–21. 10.2147/CEG.S32368 22826639 PMC3401057

[27] Gayoso-Diz P, Otero-Gonzalez A, Rodriguez-Alvarez MX, Gude F, Garcia F, De Francisco A, et al Insulin resistance (HOMA-IR) cut-off values and the metabolic syndrome in a general adult population: effect of gender and age: EPIRCE cross-sectional study. BMC Endocr Disord. 2013;13: 47 10.1186/1472-6823-13-47 24131857 PMC4016563

[28] Mazer NA. A novel spreadsheet method for calculating the free serum concentrations of testosterone, dihydrotestosterone, estradiol, estrone and cortisol: with illustrative examples from male and female populations. Steroids. 2009;74(6): 512–9. 10.1016/j.steroids.2009.01.008 19321131

[29] Kozich JJ, Westcott SL, Baxter NT, Highlander SK, Schloss PD. Development of a dual-index sequencing strategy and curation pipeline for analyzing amplicon sequence data on the MiSeq Illumina sequencing platform. Appl Environ Microbiol. 2013;79(17): 5112–20. 10.1128/AEM.01043-13 23793624 PMC3753973

[30] Edgar RC. Search and clustering orders of magnitude faster than BLAST. Bioinformatics. 2010;26(19): 2460–1. 10.1093/bioinformatics/btq461 20709691

[31] Caporaso JG, Kuczynski J, Stombaugh J, Bittinger K, Bushman FD, Costello EK, et al QIIME allows analysis of high-throughput community sequencing data. Nat Methods. 2010;7(5): 335–6. 10.1038/nmeth.f.303 20383131 PMC3156573

[32] DeSantis TZ, Hugenholtz P, Larsen N, Rojas M, Brodie EL, Keller K, et al Greengenes, a chimera-checked 16S rRNA gene database and workbench compatible with ARB. Appl Environ Microbiol. 2006;72(7): 5069–72. 10.1128/AEM.03006-05 16820507 PMC1489311

[33] Lozupone C, Knight R. UniFrac: a new phylogenetic method for comparing microbial communities. Appl Environ Microbiol. 2005;71(12): 8228–35. 10.1128/AEM.71.12.8228-8235.2005 16332807 PMC1317376

[34] Yang YW, Chen MK, Yang BY, Huang XJ, Zhang XR, He LQ, et al Use of 16S rRNA gene-targeted group-specific primers for real-time PCR analysis of predominant bacteria in mouse feces. Appl Environ Microbiol. 2015;81(19): 6749–56. 10.1128/AEM.01906-15 26187967 PMC4561689

[35] Le Chatelier E, Nielsen T, Qin J, Prifti E, Hildebrand F, Falony G, et al Richness of human gut microbiome correlates with metabolic markers. Nature. 2013;500(7464): 541–6. 10.1038/nature12506 23985870

[36] Walters WA, Xu Z, Knight R. Meta-analyses of human gut microbes associated with obesity and IBD. FEBS Lett. 2014;588(22): 4223–33. 10.1016/j.febslet.2014.09.039 25307765 PMC5050012

[37] Qin J, Li Y, Cai Z, Li S, Zhu J, Zhang F, et al A metagenome-wide association study of gut microbiota in type 2 diabetes. Nature. 2012;490(7418): 55–60. 10.1038/nature11450 23023125

[38] Karlsson FH, Tremaroli V, Nookaew I, Bergström G, Behre CJ, Fagerberg B, et al Gut metagenome in European women with normal, impaired and diabetic glucose control. Nature. 2013;498(7452): 99–103. 10.1038/nature12198 23719380

[39] Forslund K, Hildebrand F, Nielsen T, Falony G, Le Chatelier E, Sunagawa S, et al Disentangling type 2 diabetes and metformin treatment signatures in the human gut microbiota. Nature. 2015;528(7581): 262–6. 10.1038/nature15766 26633628 PMC4681099

[40] Evans CC, LePard KJ, Kwak JW, Stancukas MC, Laskowski S, Dougherty J, et al Exercise prevents weight gain and alters the gut microbiota in a mouse model of high fat diet-induced obesity. PLoS One. 2014;9(3): e92193 10.1371/journal.pone.0092193 24670791 PMC3966766

[41] Lizneva D, Suturina L, Walker W, Brakta S, Gavrilova-Jordan L, Azziz R. Criteria, prevalence, and phenotypes of polycystic ovary syndrome. Fertil Steril. 2016;pii: S0015. 10.1016/j.fertnstert.2016.05.003 27233760

[42] Kelly BJ, Gross R, Bittinger K, Sherril-Mix S, Lewis JD, Collmann RG, et al Power and sample-size estimation for microbiome studies using pairwise distances and PERMANOVA. Bioinformatics. 2015;31(15): 2461–8. 10.1093/bioinformatics/btv183 25819674 PMC4514928

[43] Brown ZA, Louwers Y V, Fong SL, Valkenburg O, Birnie E, de Jong FH, et al The phenotype of polycystic ovary syndrome ameliorates with aging. Fertil Steril. 2011;96(5): 1259–65. 10.1016/j.fertnstert.2011.09.002 21963227

[44] Farkas AM, Panea C, Goto Y, Nakato G, Galan-Diez M, Narushima S, et al Induction of Th17 cells by segmented filamentous bacteria in the murine intestine. J Immunol Methods. 2015;421: 104–11. 10.1016/j.jim.2015.03.020 25858227 PMC4764030

[45] Yin Y, Wang Y, Zhu L, Liu W, Liao N, Jiang M, et al Comparative analysis of the distribution of segmented filamentous bacteria in humans, mice and chickens. ISME J. 2013;7(3): 615–21. 10.1038/ismej.2012.128 23151642 PMC3578561

[46] Sapone A, de Magistris L, Pietzak M, Clemente MG, Tripathi A, Cucca F, et al Zonulin upregulation is associated with increased gut permeability in subjects with type 1 diabetes and their relatives. Diabetes. 2006;55(5): 1443–9. 16644703 10.2337/db05-1593

[47] Zak-Golab A, Kocelak P, Aptekorz M, Zientara M, Juszczyk L, Martirosian G, et al Gut microbiota, microinflammation, metabolic profile, and zonulin concentration in obese and normal weight subjects. Int J Endocrinol. 2013;2013: 674106 10.1155/2013/674106 23970898 PMC3732644

