# Supplementary figures and images for: Alterations in Gut Microbiome Composition and Barrier Function Are Associated with Reproductive and Metabolic Defects in Women with Polycystic Ovary Syndrome (PCOS): A Pilot Study
Source: PLoS One. 2017 Jan 3;12(1):e0168390. doi: 10.1371/journal.pone.0168390 (PMC5207627; doi:10.1371/journal.pone.0168390)

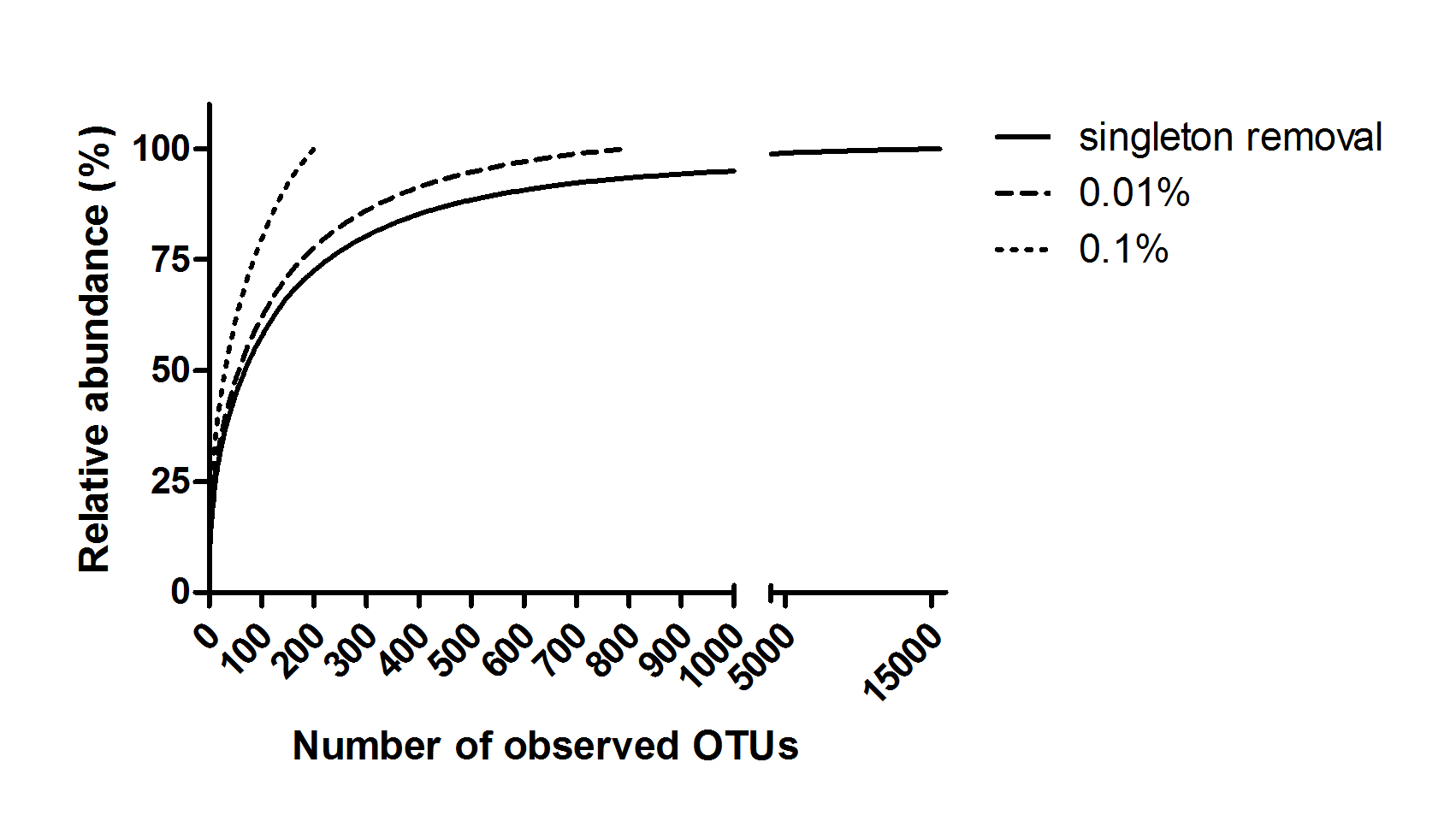

Supplement: S1 Fig — (JPG) [file pone.0168390.s001.jpg]

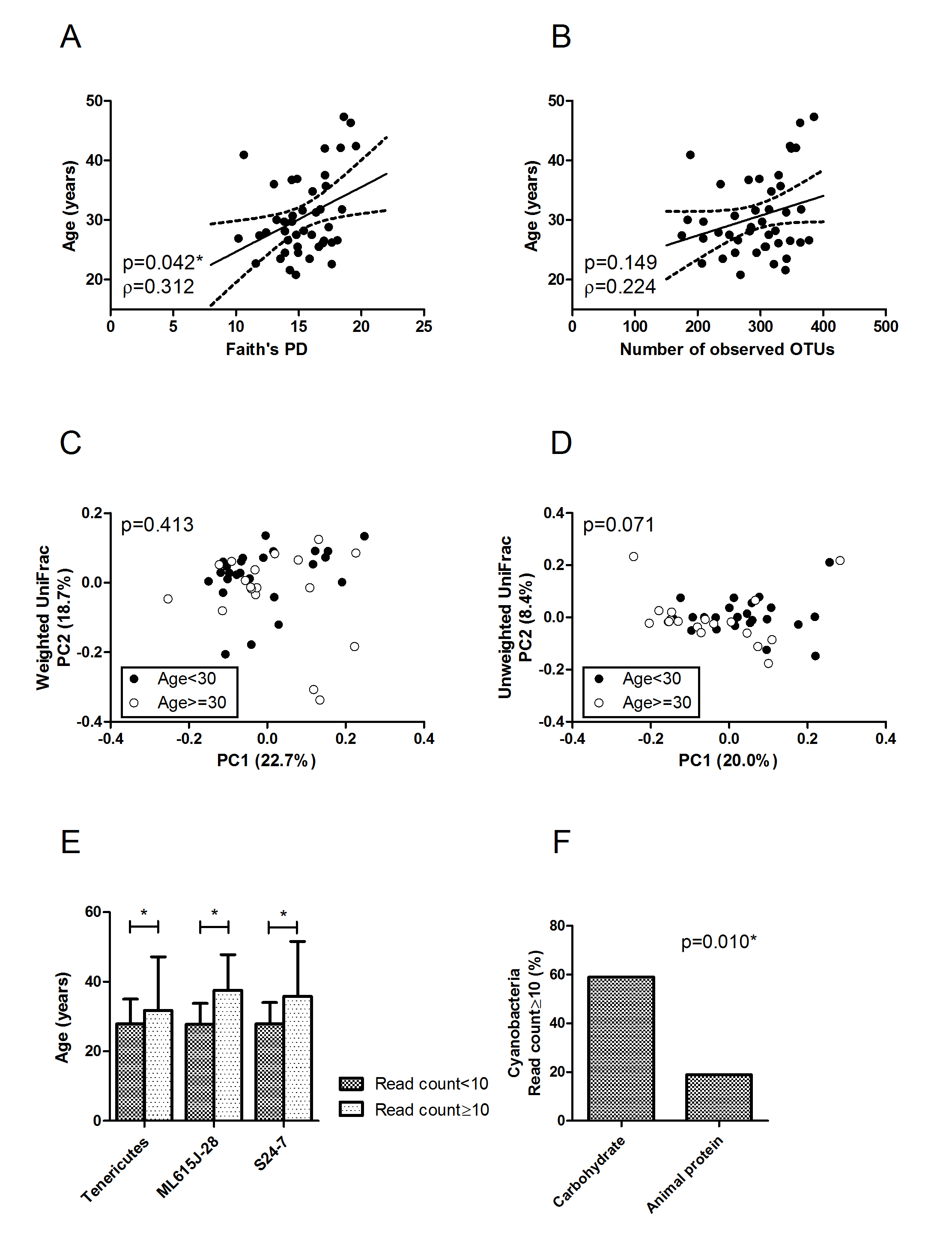

Supplement: S2 Fig — (JPG) [file pone.0168390.s002.jpg]

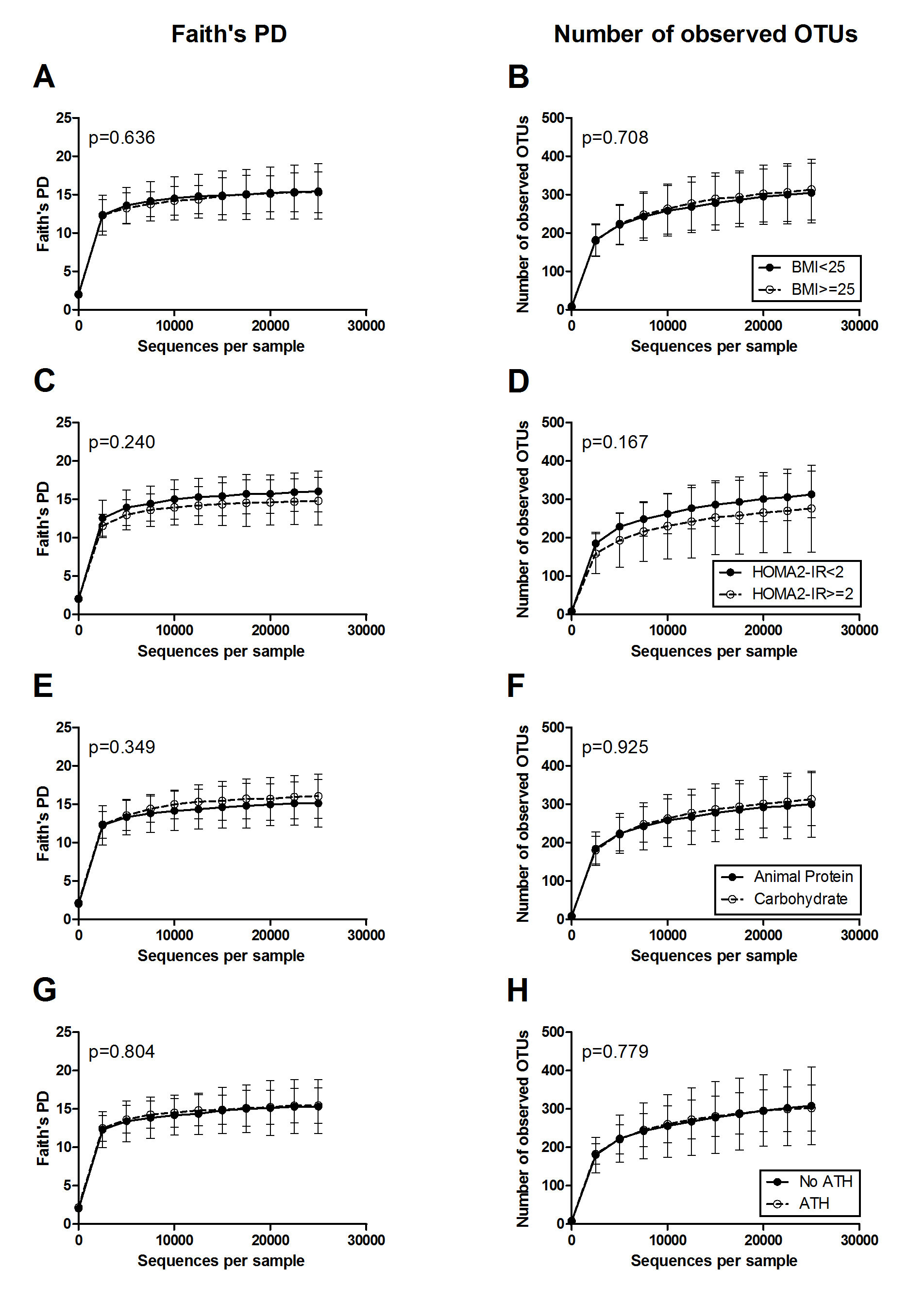

Supplement: S3 Fig — ATH: adult-type hypolactasia. (JPG) [file pone.0168390.s003.jpg]

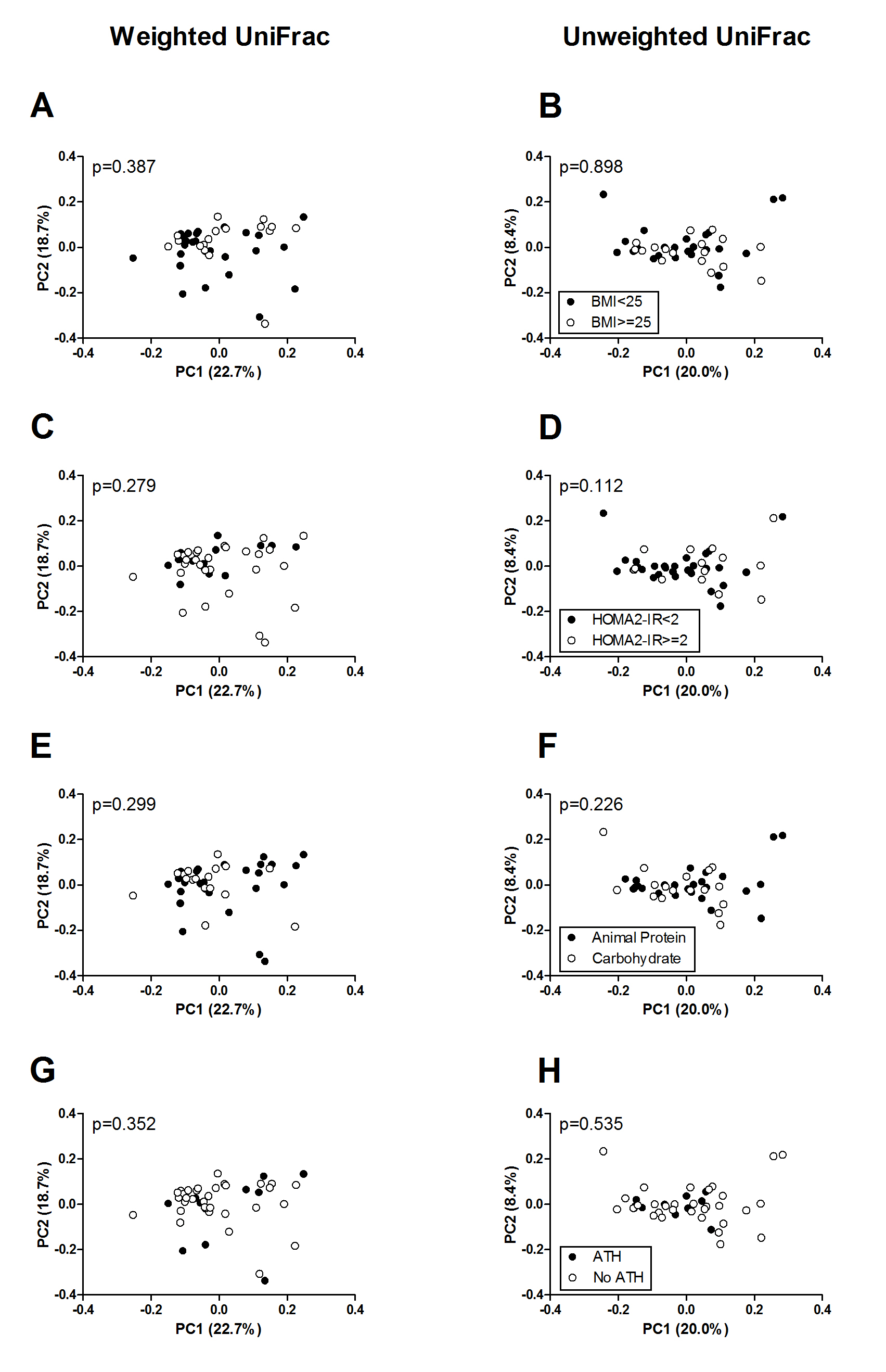

Supplement: S4 Fig — ATH: adult-type hypolactasia. (JPG) [file pone.0168390.s004.jpg]
