# Supplementary material for: Alterations in Gut Microbiome Composition and Barrier Function Are Associated with Reproductive and Metabolic Defects in Women with Polycystic Ovary Syndrome (PCOS): A Pilot Study
Source: PLoS One. 2017 Jan 3;12(1):e0168390. doi: 10.1371/journal.pone.0168390 (PMC5207627; doi:10.1371/journal.pone.0168390)
Supplement: S1 File — (PDF) [file pone.0168390.s005.pdf]

Supplementary Materials and Methods

Study cohort

PCOS was diagnosed according to the Rotterdam Criteria, which require the presence of two out of three of the following symptoms: clinical/biochemical hyperandrogenism, oligo-/anovulation, and polycystic ovaries. Additionally, other disorders which may mimic the PCOS phenotype were excluded [1].

Clinical hyperandrogenism was assessed using the modified Ferriman-Gallwey (FG) Score, with a self-reported score of eight or higher indicating hirsutism [2]. Biochemical hyperandrogenism was defined according to the recommendations of the European Society of Human Reproduction and Embryology and the Androgen Excess Society based on serum values of total testosterone and dehydroepiandrosterone sulfate (DHEAS) [1,3]. For total testosterone, a cut-off of 2.1 nmol/l was used based on previously published data from a representative population sample [4]. As there is currently no reference range available for serum DHEAS assessed by mass spectrometry, a cut-off of 7.5 µmol/l was chosen based on the empirically determined reference range of the endocrine laboratory at the Department of Endocrinology and Diabetology, University Hospital Graz. As DHEAS is routinely measured by immunoassay, which typically overestimates concentrations due to cross-reactivity, this cut-off is considered conservative [5]. Oligo-/anovulation was defined as menstrual cycles with a duration >35 days or the absence of menstruation for three or more consecutive months. Polycystic ovarian morphology, diagnosed by a gynecological ultrasound, was assessed based on medical history. Thyroid disorder, congenital adrenal hyperplasia, Cushing's syndrome, hyperprolactinemia, androgen-secreting tumors, and pregnancy were excluded by laboratory measurements of thyroid-stimulating hormone (TSH), 17-hydroxyprogesterone (17OH-P), cortisol, prolactin, pregnancy test, and clinical examination. Healthy controls did not meet any of the Rotterdam Criteria, with the following exceptions: isolated elevation of DHEAS or androstenedione (6 subjects) and long-standing hirsutism (1 subject), as these features are not considered pathological in the absence of reproductive or cosmetic symptoms of hyperandrogenism [1].

Next-generation sequencing

Total DNA was extracted from stool samples using the MagNA Pure LC DNA Isolation Kit III (Bacteria, Fungi) on the MagNA Pure Instrument (Roche, Rotkreuz, Switzerland). Stool was thawed partially and an amount approximately the size of a maize kernel was homogenized in 500 µl 1x phosphate buffered saline (PBS). 250 µl of the diluted sample was added to 250 µl bacteria lysis buffer in a sample tube containing MagNA Lyser Green Beads (1.4 mm diameter ceramic beads, Roche). Samples were homogenized in a MagNA Lyser Instrument (2 x 6000 rpm for 30 s, separated by 1 min cooling), treated with 25 µl lysozyme (Roth, Karlsruhe, Germany) at 37 ^{0}C for 30 minutes, and then with 43.3 µl proteinase K (Roche) at 60 ^{0}C for 1 hour. Lysates were incubated at 95 ^{0}C for 10 minutes, cooled on ice for 5 minutes, and centrifuged for 5 minutes at full speed. DNA was isolated from 100 µl lysate supernatant by the MagNA Pure Instrument using the manufacturer's software and eluted in 100 µl elution buffer. A PCR reaction was performed to amplify the V1-2 region of the bacterial 16S rRNA gene using the primers F27 (AGAGTTTGATCCTGGCTCAG) and R357 (CTGCTGCCTYCCGTA) (Eurofins Genomics, Ebersberg, Germany) and the FastStart High Fidelity PCR System, dNTPack (Roche) with initial denaturation at 95 °C for 3 minutes followed by 28 cycles of denaturation at 95 °C for 45 seconds, annealing at 55 °C for 45 seconds, and extension at 72 °C for 1 minute, one cycle of final extension at 72 °C for 7 minutes, and a final cooling step to 10 °C. Triplicates were pooled, checked on a 1.5 % agarose gel, and 15 µl of pooled PCR product were normalized according to manufacturer's instructions on a SequalPrep Normalization Plate (Life Technologies, Vienna, Austria). 15 µl of the normalized PCR product were used as a template for indexing PCR in a 50 µl single reaction to introduce barcode sequences to each sample according to Kozich *et al.* [6]. Cycling conditions were initial denaturation at 95 °C for 3 minutes followed by 8 cycles of denaturation at 95 °C for 45 seconds, annealing at 55 °C for 45 seconds, and extension at 72 °C for 1 minute, one cycle of final extension at 72 °C for 7 minutes, and a final cooling step to 4 °C. After indexing, 5 µl of each sample were pooled and 50 µl of the unpurified library were loaded on a 1 % agarose gel and purified from the gel with the Qiaquick Gel Extraction Kit (Qiagen, Hilden, Germany) according to the manufacturer's instructions. The pooled DNA was quantified using QuantiFluor ONE dsDNA dye on a Quantus Fluorometer (Promega, Mannheim, Germany) according

to the manufacturer’s instructions and visualized for size validation on a 2100 Bioanalyzer Instrument (Agilent Technologies, Santa Clara, USA) using a high-sensitivity DNA assay according to the manufacturer’s instructions. The final 6 pM library containing all pooled samples was run with 20 % PhiX and v3, 600 cycles chemistry according to the manufacturer's instructions on a MiSeq desktop sequencer (Illumina, Eindhoven, Netherlands).

Sequencing data analysis

Raw reads were processed using the open-source software mothur V1.35.0 according to the protocol by Kozich *et al.* (accessed April 2015), with the following adaptations: no maxlength was defined during the screening step, start (1,046) and end (6,426) positions were adapted to the V1-V2 region, and a difference of three bases was permitted during the precluster step (based on the recommendation by the authors to allow one mismatch per 100 bp) [6]. Chimeric sequences were identified by UCHIME and subsequently removed [7]. After removal of non-bacterial sequences, classified using the SILVA 119 database (www.arb-silva.de), the remaining sequences were degapped, deuniqued, split into individual samples, and formatted for use with the open-source software QIIME 1.8.0 [8].

Statistical analysis

The following variables contained missing values: AMH (2 control, 4 PCOS); LH, LH:FSH ratio, hsCRP (1 control, 1 PCOS each); HDL-cholesterol, polycystic ovarian morphology (1 PCOS each); DHT, DHEAS, free testosterone, free DHT, stool calprotectin, stool zonulin (1 control each).

Supplementary References

1. Rotterdam ESHRE/ASRM-Sponsored PCOS consensus Workshop Group. Revised 2003 consensus on diagnostic criteria and long-term health risks related to polycystic ovary syndrome (PCOS). Hum Reprod. 2004;19(1): 41–7.

2. Yildiz BO, Bolour S, Woods K, Moore A, Azziz R. Visually scoring hirsutism . Hum Reprod Update.

2010;16(1): 51–64.

3. Azziz R, Carmina E, Dewailly D, Diamanti-Kandarakis E, Escobar-Morreale HF, Futterweit W, et al. Criteria for Defining Polycystic Ovary Syndrome as a Predominantly Hyperandrogenic Syndrome: An Androgen Excess Society Guideline. J Clin Endocrinol Metab. 2006;91(11): 4237.

4. Haring R, Hannemann A, John U, Radke D, Nauck M, Wallaschofski H, et al. Age-specific reference ranges for serum testosterone and androstenedione concentrations in women measured by liquid chromatography-tandem mass spectrometry . J Clin Endocrinol Metab. 2012;97(2): 408–15.

5. Chadwick CA, Owen LJ, Keevil BG. Development of a method for the measurement of dehydroepiandrosterone sulphate by liquid chromatography-tandem mass spectrometry . Ann Clin Biochem. 2005;42(Pt 6): 468–74.

6. Kozich JJ, Westcott SL, Baxter NT, Highlander SK, Schloss PD. Development of a dual-index sequencing strategy and curation pipeline for analyzing amplicon sequence data on the MiSeq Illumina sequencing platform . Appl Environ Microbiol. 2013;79(17): 5112–20.

7. Edgar RC, Haas BJ, Clemente JC, Quince C, Knight R. UCHIME improves sensitivity and speed of chimera detection . Bioinformatics. 2011;27(16): 2194–200.

8. Caporaso JG, Kuczynski J, Stombaugh J, Bittinger K, Bushman FD, Costello EK, et al. QIIME allows analysis of high-throughput community sequencing data . Nat Methods. 2010;7(5): 335–6.
