# Supplementary material for: Alterations in Gut Microbiome Composition and Barrier Function Are Associated with Reproductive and Metabolic Defects in Women with Polycystic Ovary Syndrome (PCOS): A Pilot Study
Source: PLoS One. 2017 Jan 3;12(1):e0168390. doi: 10.1371/journal.pone.0168390 (PMC5207627; doi:10.1371/journal.pone.0168390)
Supplement: S2 File — (PDF) [file pone.0168390.s006.pdf]

Landeskrankenhaus - Universitätsklinikum Graz

Ernährungsmedizinischer Dienst

Ltd. Diaetologin: Eisenberger Anna Maria

A-8036 Graz, Auenbruggerplatz 21, Telefon: +43 (316) 385-82958, Fax: +43 (316) 385-4745

Steiermärkische Krankenanstaltengesellschaft m.b.H. *Medizinische Universität Graz*

StudienID

PatientenID

Ernährungsfragebogen

Datum:

Wie häufig verzehren Sie folgende Lebensmittel und Getränke?

**Getreideprodukte**	mehrmals täglich	täglich	mehrmals pro Woche	etwa 1 x pro Woche	mehrmals pro Monat	seltener/ nie
Brot						
Müsli						
Vollkornprodukte						
Kartoffeln, Reis, Nudeln						
**Milch und Milchprodukte**	mehrmals täglich	täglich	mehrmals pro Woche	etwa 1 x pro Woche	mehrmals pro Monat	seltener/ nie
Käsesorten mind. 45% Fett i. Tr.						
fettarme Käsesorten mit weniger als 45% Fett i. Tr.						
Milch, Topfen, Joghurt o.ä. mit 3,5% Fett						
Milch, Topfen, Joghurt o.ä. mit 1,5%Fett						
Sauerrahm, Obers, Creme fraîche						
**Fleisch- und Fischprodukte**	mehrmals täglich	täglich	mehrmals pro Woche	etwa 1 x pro Woche	mehrmals pro Monat	seltener/ nie
Fleisch						
Fettreiche Wurstsorten (z.B. Leberstreichwurst, Salami, Bergsteiger, Mortadella, …)						
Fettarme Wurstsorten (z.B. Putenwurst, Schinken, Krakauer,…)						
Fisch						
Fischkonserven						
**Obst und Gemüse**	mehrmals täglich	täglich	mehrmals pro Woche	etwa 1 x pro Woche	mehrmals pro Monat	seltener/ nie
Gemüse und Salat						
Obst						

**Fette**	mehrmals täglich	täglich	mehrmals pro Woche	etwa 1 x pro Woche	mehrmals pro Monat	seltener/ nie
Butter, Margarine						
Halbfettbutter oder -margarine						
Öle						
Schmalz						
**Extras**	mehrmals täglich	täglich	mehrmals pro Woche	etwa 1 x pro Woche	mehrmals pro Monat	seltener/ nie
Süßigkeiten, Schokolade						
Kuchen, Kekse, Torten						
Eis, Pudding und süße Speisen						
In Fett gebackene Speisen (Wiener Schnitzel, Pommes frites,….)						
Chips, Erdnüsse, Knabbereien						
**Getränke**	mehrmals täglich	täglich	mehrmals pro Woche	etwa 1 x pro Woche	mehrmals pro Monat	seltener/ nie
Mineralwasser, verdünnte Obstsäfte, Wasser, Tee						
Limonaden, Cola						
Limonaden oder Cola mit Süßstoff						
Obst- und Gemüsesäfte unverdünnt						
Kakao, Milch, Milchshakes						
Kaffee						
Bier, Wein, sonstige alkoholische Getränke						
Energiedrinks						
**Sojaprodukte**	mehrmals täglich	täglich	mehrmals pro Woche	etwa 1 x pro Woche	mehrmals pro Monat	seltener/ nie
Sojamehl, Sojabohnen						
Tofu, Tempeh, Miso, Natto, Sojasprossen						
Fleischersatzprodukte mit Soja (Sojaburger, Sojawürstchen etc.)						
Milchersatzprodukte mit Soja (Sojamilch, Drinks, Joghurt, Dessert, Käse, Eis etc.)						
Nahrungsergänzungsmittel mit Soja oder Isoflavonen						
Andere:						

Landeskrankenhaus - Universitätsklinikum Graz

Ernährungsmedizinischer Dienst

Ltd. Diaetologin: Eisenberger Anna Maria

A-8036 Graz, Auenbruggerplatz 21, Telefon: +43 (316) 385-82958, Fax: +43 (316) 385-4745

Steiermärkische Krankenanstaltengesellschaft m.b.H. *Medizinische Universität Graz*

Food Frequency Questionnaire

StudyID

PatientID

Date:

How often do you consume the following foods and beverages?

**Grains**	Multiple times a day	Daily	Multiple times a week	About once a week	Multiple times a month	Less often/ never
Bread						
Muesli						
Whole wheat products						
Potatoes, rice, pasta						
**Milk and Dairy**	Multiple times a day	Daily	Multiple times a week	About once a week	Multiple times a month	Less often/ never
Cheese, min. 45% fat content						
Low-fat cheese, <45% fat content						
Milk, curd, yoghurt, or similar, 3,5% Fett						
Milk, curd, yoghurt, or similar, 1,5%Fett						
Sour cream, cream, creme fraîche						
**Meat and Fish**	Multiple times a day	Daily	Multiple times a week	About once a week	Multiple times a month	Less often/ never
Meat						
Fatty cold cuts (e.g. liverwurst, salami, mortadella, …)						
Low-fat cold cuts (e.g. turkey, ham,…)						
Fish						
Canned fish						
**Fruits and Vegetables**	Multiple times a day	Daily	Multiple times a week	About once a week	Multiple times a month	Less often/ never
Vegetables and salad						
Fruits						
**Fats**	Multiple times a	Daily	Multiple times a	About once a	Multiple times a	Less often/

	day		week	week	month	never
Butter, margarine						
Low-fat butter or margarine						
Oils						
Lard						
**Extras**	Multiple times a day	Daily	Multiple times a week	About once a week	Multiple times a month	Less often/ never
Sweets, chocolate						
Cake, cookies						
Ice cream, pudding						
Deep-fried foods (Wiener Schnitzel, french fries,….)						
Chips, peanuts, salty snacks						
**Drinks**	Multiple times a day	Daily	Multiple times a week	About once a week	Multiple times a month	Less often/ never
Mineral water, diluted fruit juices, water, tea						
Lemonade, soda						
Sugar-free lemonade or soda						
Undiluted fruit and vegetable juices						
Cocoa, milk, milkshakes						
Coffee						
Beer, wine, other alcoholic beverages						
Energy drinks						
**Soy Products**	Multiple times a day	Daily	Multiple times a week	About once a week	Multiple times a month	Less often/ never
Soy meal, soy beans						
Tofu, tempeh, miso, natto, soy sprouts						
Meat replacements with soy (soy burger, soy sausage etc.)						
Dairy replacements with soy (soy milk, drinks, yoghurt, dessert, cheese, ice cream etc.)						
Supplements containing soy or isoflavones						
Others:						
