# Supplementary material for: Alterations in Gut Microbiome Composition and Barrier Function Are Associated with Reproductive and Metabolic Defects in Women with Polycystic Ovary Syndrome (PCOS): A Pilot Study
Source: PLoS One. 2017 Jan 3;12(1):e0168390. doi: 10.1371/journal.pone.0168390 (PMC5207627; doi:10.1371/journal.pone.0168390)
Supplement: S1 Table — (DOCX) [file pone.0168390.s008.docx]

**S1 Table. Detection limits and reliability indices of used assays**

| **Analyte** | **Assay type** | **Detection limit** | **Intra-assay CV (%)** | **Inter-assay CV (%)** |
| --- | --- | --- | --- | --- |
| E1 | LC-MS-MS | 3.9 pmol/l | 4.7 | 2.1-3.4 |
| E2 | LC-MS-MS | 8.0 pmol/l | 6.7 | 3.4-5.0 |
| Total testosterone | LC-MS-MS | 0.3 nmol/l | <10 | <6 |
| Androstenedione | LC-MS-MS | 0.3 nmol/l | <10 | <7 |
| DHEA | LC-MS-MS | 1 nmol/l | <10 | <10 |
| DHEAS | LC-MS-MS | 1 μmol/l | 2.9-11.3 | 3.7-7.3 |
| DHT | LC-MS-MS | 0.3 nmol/l | <10 | <10 |
| Cortisol | chemiluminescence immunoassay, ADVIA Centaur XP (Roche) | 3.9 nmol/l | 2.9-4.2 | 4.4-6.0 |
| TSH | chemiluminescence immunoassay, ADVIA Centaur XP (Roche) | 0.01 mU/l | 2.4-2.5 | 2.1-5.3 |
| Prolactin | chemiluminescence immunoassay, ADVIA Centaur XP (Roche) | 6.4 mU/l | 1.9-4.4 | 2.0-5.3 |
| Insulin | chemiluminescence immunoassay, ADVIA Centaur XP (Roche) | 0.5 mU/l | 3.2-4.6 | 2.6-5.9 |
| AMH | chemiluminescence immunoassay, Access2 (Beckman Coulter) | 0.1 pmol/l | 0-1.7 | 0-3.1 |
| SHBG | chemiluminescence immunoassay, Cobas e411 (Roche) | 0.35 nmol/l | 2.1-2.7 | 2.7-5.6 |
| LH | ELISA (DiaSource) | 1.3 IU/l | 4.5-7.6 | 3.2-11.0 |
| FSH | ELISA (DiaSource) | 0.9 IU/l | 4.2-7.9 | 5.2-7.2 |
| 17OH-P | ELISA (IBL International) | 0.1 nmol/l | 2.8-4.9 | 5.8-9-2 |
| hs-CRP | ELISA (BioVendor) | 0.02 mg/l | 4.1-6.9 | 5.8-6.3 |
| IL-6 | ELISA (BioVendor) | 0.9 pg/ml | 0.2-7.8 | 0-17.8 |
| TNF-α | ELISA (BioVendor) | 2.3 pg/ml | 1.9-10.4 | 4.5-10.3 |
| Zonulin (serum) | ELISA (Immundiagnostik) | 0.2 ng/ml | 3.4-6.0 | 13.3-13.6 |
| Zonulin (stool) | ELISA (Immundiagnostik) | 0.2 ng/ml | 3.2-6.2 | 12.7-13.9 |
| DAO | ELISA (Immundiagnostik) | 0.12 U/m | 1.4-1.7 | 7.9-10.7 |
| LBP | ELISA (Hycult Biotech) | 4.4 ng/ml | 0.05-8.5 | § |
| sCD14 | ELISA (R&D Systems) | 125 pg/mL | 4.8-6.4 | 4.8-7.4 |
| Calprotectin | ELISA (Bühlmann Laboratories) | 10 µg/g | 2.7-8.1 | 6.6-14.5 |
| Total cholesterol | Enzymatic colorimetric assay, Cobas c (Roche) | 0.1 mmol/l | 0.6 | 1.4-1.6 |
| HDL-cholesterol | Enzymatic colorimetric assay, Cobas c (Roche) | 0.1 mmol/l | 0.5-0.7 | 0.9 |
| Triglycerides | Enzymatic colorimetric assay, Cobas c (Roche) | 0.1 mmol/l | 0.7 | 1.9 |
| Glucose | Enzymatic colorimetric assay, Cobas c (Roche) | 0.1 mmol/l | 0.5-0.7 | 1.1-1.2 |
| LPS | HEK-Blue LPS detection kit (Invivogen) | 0.01 EU/ml | § | § |

CV: coefficient of variation; §reference range not defined.
