# Supplementary material for: Alterations in Gut Microbiome Composition and Barrier Function Are Associated with Reproductive and Metabolic Defects in Women with Polycystic Ovary Syndrome (PCOS): A Pilot Study
Source: PLoS One. 2017 Jan 3;12(1):e0168390. doi: 10.1371/journal.pone.0168390 (PMC5207627; doi:10.1371/journal.pone.0168390)
Supplement: S2 Table — (DOCX) [file pone.0168390.s009.docx]

**S2 Table. Overview of sequencing data**

|  | **Number of reads analyzed** | | | | | **Number of OTUs detected** | | | | |
| --- | --- | --- | --- | --- | --- | --- | --- | --- | --- | --- |
|  | **Control (n=19)** | | **PCOS (n=24)** | |  | **Control (n=19)** | | **PCOS (n=24)** | |  |
| **Cut-off** | **mean** | **SD** | **mean** | **SD** | **p-value** | **mean** | **SD** | **mean** | **SD** | **p-value** |
| Singleton removal | 65249 | 16528.8 | 65462 | 10860.4 | 0.962 | 974 | 145.6 | 894 | 149.7 | 0.092 |
| >0.01 % | 60719 | 16075.3 | 61472 | 10207.3 | 0.860 | 315 | 52.3 | 279 | 54.6 | 0.030* |
| >0.1 % | 46449 | 15512.8 | 48639 | 8006.6 | 0.581 | 106 | 17.0 | 95 | 16.8 | 0.062 |

Number of reads analyzed and OTUs detected at three relative abundance cut-off levels. OTU: operational taxonomic unit, SD: standard deviation.
