# Supplementary material for: Alterations in Gut Microbiome Composition and Barrier Function Are Associated with Reproductive and Metabolic Defects in Women with Polycystic Ovary Syndrome (PCOS): A Pilot Study
Source: PLoS One. 2017 Jan 3;12(1):e0168390. doi: 10.1371/journal.pone.0168390 (PMC5207627; doi:10.1371/journal.pone.0168390)
Supplement: S3 Table — (DOCX) [file pone.0168390.s010.docx]

**S3 Table. Contaminations**

|  | **# of reads in NCs (n=8)** | |
| --- | --- | --- |
| **Genus** | **median** | **IQR** |
| *Comamonadaceae unclass. | 1326 | 738.5 |
| *Pseudomonas | 256 | 235.0 |
| *Enterobacteriaceae unclass. | 154 | 130.5 |
| †Bacteroides | 123 | 685.5 |
| ‡Alkalibacterium | 92 | 137.5 |
| *Delftia | 59 | 79.5 |
| *Xanthomonadaceae unclass. 1 | 38 | 67.5 |
| *Stenotrophomonas | 25 | 58.5 |
| *Caulobacteraceae unclass. | 20 | 35.0 |
| §Microbacterium | 14 | 31.0 |
| *Neisseriaceae unclass. | 11 | 33.5 |
| ‡Streptococcus | 11 | 29.0 |
| *Oceanospirillales unclass. | 5 | 11.5 |
| \|\|Fimbriimonas | 5 | 24.5 |
| *Aeromonadaceae unclass. | 4 | 13.5 |
| *Acinetobacter | 4 | 13.0 |
| *Neisseria | 3 | 21.5 |
| §Proprionibacterium | 3 | 37.5 |
| †Pedobacter | 2 | 24.5 |
| *Ochrobactrum | 1 | 17.5 |
| *Gluconacetobacter | 1 | 8.0 |
| †Prevotella | 1 | 3.0 |
| †Bacteroidales unclass. | 0 | 43.0 |
| §Corynebacterium | 0 | 11.5 |
| §Rothia | 0 | 0.5 |
| *Bradyrhizobium | 0 | 18.0 |
| *Alteromonadales unclass. | 0 | 11.5 |
| ‡Staphylococcus | 0 | 0.5 |
| ‡Acidaminococcus | 0 | 9.5 |
| *Haemophilus | 0 | 7.0 |
| ¶Leptotrichia | 0 | 0.5 |
| ‡Clostridium | 0 | 12.0 |
| *Sulfurospirillum | 0 | 12.0 |
| ‡Granulicatella | 0 | 9.0 |
| †Chryseobacterium | 0 | 4.0 |
| ¶Fusobacterium | 0 | 0.5 |
| §Actinomycetales unclass. | 0 | 3.0 |
| †Barnesiellaceae unclass. | 0 | 1.0 |
| *Xanthomonadaceae unclass. 2 | 0 | 4.0 |
| *Shewanella | 0 | 4.5 |
| *Sphingomonadaceae unclass. | 0 | 3.0 |
| *Oxalobacteraceae unclass. | 0 | 4.0 |
| ‡Lactobacillus | 0 | 0.5 |
| ‡Listeria | 0 | 1.0 |
| *Campylobacter | 0 | 0.5 |
| *Enhydrobacter | 0 | 2.0 |
| ‡Veillonella | 0 | 1.0 |
| **F16 unclass. | 0 | 0.5 |
| ‡Enterococcaceae unclass. | 0 | 0.5 |
| *Vibrio | 0 | 1.0 |
| ‡Lachnospiraceae unclass. | 0 | 0.5 |
| ‡Clostridiales unclass. | 0 | 0.5 |

Read counts of bacterial genera which were detected in at least two negative controls (NCs). unclass.: unclassified, the lowest classified taxonomic level is shown. *Proteobacteria, †Bacteroidetes, ‡Firmicutes, §Actinobacteria, ||Armatimonadetes, ¶Fusobacteria, **TM7.
